# Well-differentiated endometrial adenocarcinoma demonstrating morphological spectrum: a clinical image

**DOI:** 10.11604/pamj.2026.53.142.50774

**Published:** 2026-03-25

**Authors:** Shubham Kalode, Punam Sawarkar

**Affiliations:** 1 Department of Panchakarma, Mahatma Gandhi Ayurved College, Hospital and Research Centre, Datta Meghe Institute of Higher Education and Research, Salod (H), Wardha, Maharashtra, India

**Keywords:** Endometrial adenocarcinoma, well differentiated, clinical image

## Image in medicine

Endometrial adenocarcinoma is the most common malignancy of the female genital tract, predominantly affecting postmenopausal women. A 58-year-old postmenopausal woman presented with abnormal uterine bleeding for three months. Transvaginal ultrasonography revealed a thickened endometrium. An endometrial biopsy was performed, which was suggestive of malignancy. The patient subsequently underwent total abdominal hysterectomy with bilateral salpingo-oophorectomy. Gross examination of the specimen revealed an irregular gray-white friable growth measuring 3 x 2 cm confined to the endometrial cavity. Histopathological examination showed closely packed back-to-back glands lined by tall columnar epithelial cells resembling proliferative endometrium. The nuclei exhibited mild enlargement with minimal atypia and inconspicuous nucleoli. Mitotic figures were infrequent, and the intervening stroma was scant. No definite myometrial or lymphovascular invasion was identified. These findings were consistent with well-differentiated (grade 1) endometrioid adenocarcinoma of the endometrium. A hysterectomy was conducted. The postoperative period was uneventful. The patient was advised on regular follow-up, and no evidence of disease progression was noted during the short-term follow-up period.

**Figure 1:**
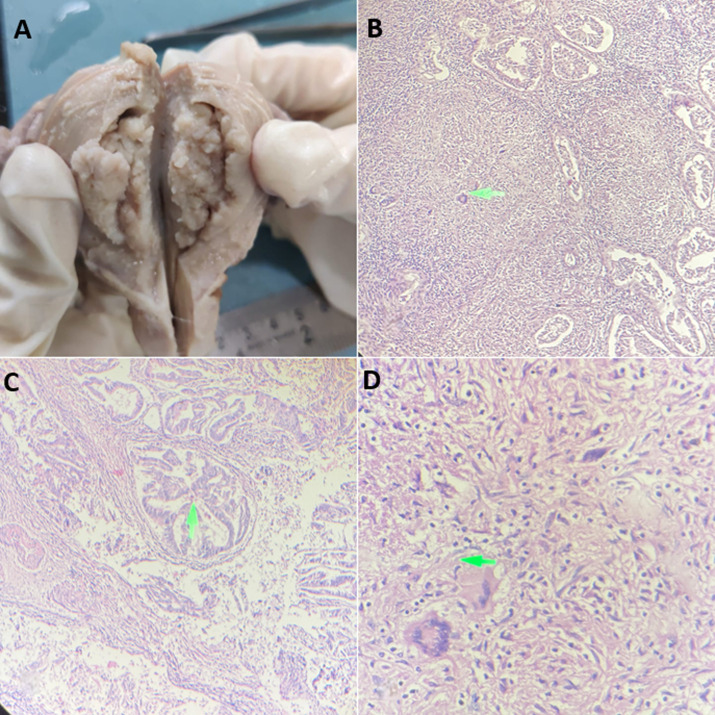
A) gross image of hysterectomy specimen showing an irregular gray-white friable growth involving the endometrial cavity; B) low-power (10X) photomicrograph (H&E) showing closely packed back-to-back glands with scant intervening stroma; C) low-power(10X) photomicrograph (H&E) demonstrating complex glandular architecture with preserved endometrioid morphology; D) high-power view (40X, H&E) showing glands lined by tall columnar cells with mild nuclear atypia and infrequent mitoses, consistent with well differentiated (grade 1) endometrial adenocarcinoma (green arrows: complex glandular architecture with nuclear atypia and infrequent mitoses)

